# BRDF-Corrected Vicarious Calibration of FORMOSAT-5 RSI Using RadCalNet: Quantitative Assessment and Implications for TOA Reflectance and NIRv

**DOI:** 10.3390/s26123719

**Published:** 2026-06-11

**Authors:** Yi-Ling Chang, Kuo-En Chang, Kuo-Hsien Hsu, Liang-De Chen, Nguyen Van Hieu, Tang-Huang Lin

**Affiliations:** 1 Department of Space Science and Engineering, National Central University, Taoyuan City 320317, Taiwan; yiling@csrsr.ncu.edu.tw; 2 Center for Space and Remote Sensing Research, National Central University, Taoyuan City 320317, Taiwan; 3 Center for Environmental Monitoring and Technology, National Central University, Taoyuan City 320317, Taiwan; 4 Taiwan Space Agency (TASA), Hsinchu City 300091, Taiwan; 5 Geoinformatics Research Center, Thai Nguyen University of Agriculture and Forestry, Thai Nguyen City 250000, Vietnam

**Keywords:** FORMOSAT-5, vicarious calibration, radiometric calibration coefficient, RadCalNet, BRDF correction, NIRv, 6S radiative transfer model

## Abstract

**Highlights:**

**What are the main findings?**

- Incorporating an empirical BRDF lookup table from in situ multi-angle measurements at Railroad Valley Playa reduced the $K_{0}$ relative error from 13–17% to 1–4% across all five FORMOSAT-5 spectral bands.
- The calibration improvement propagates to a scene-mean 7.88% relative difference in NIRv over a heterogeneous La Crau scene, with localized differences exceeding 20% in densely vegetated areas.

**What are the implications of the main findings?**

- BRDF correction is an operational prerequisite for retrieving reliable vegetation products from FORMOSAT-5, and similar high-resolution optical missions lack comprehensive onboard calibration.
- The proposed framework provides quantitative calibration-accuracy requirements for vicarious calibration of off-nadir satellite observations, with direct relevance to applications of carbon cycles and ecosystem monitoring.

**Abstract:**

Accurate radiometric calibration is essential for high-resolution optical satellite sensors with limited onboard calibration capability, such as the FORMOSAT-5 (FS-5) Remote Sensing Instrument (RSI). The Radiometric Calibration Network (RadCalNet) provides standardized nadir-equivalent surface reflectance for vicarious calibration, but its direct application to off-nadir observations can introduce systematic biases over non-Lambertian surfaces. This study presents a BRDF-corrected vicarious calibration framework for the FS-5 RSI. The framework integrates RadCalNet data with an empirical BRDF lookup table built from in situ multi-angle measurements at Railroad Valley Playa, which is then propagated through 6S radiative transfer simulation. Applied to four FS-5 overpasses, BRDF correction reduced the median relative error of the calibration coefficient $K_{0}$ from 13–17% to 1–4% across all five spectral bands, providing a quantitative assessment of calibration improvement. The downstream impact was evaluated over an FS-5 La Crau scene. Scene-mean top-of-atmosphere (TOA) reflectance differences across the four multispectral bands ranged from 8.62% (NIR) to 10.99% (Green). The near-infrared reflectance of vegetation (NIRv), a proxy for gross primary production, showed a scene-mean relative difference of 7.88% ± 7.32%, with localized values exceeding 20% in densely vegetated areas. These results establish quantitative calibration-accuracy requirements for sensors relying on vicarious calibration and demonstrate the operational necessity of BRDF correction for reliable TOA reflectance and vegetation product retrieval.

## 1. Introduction

After launch, optical satellite sensors experience continuous radiometric degradation due to thermal cycling and radiation exposure in orbit [1,2,3,4]. This degradation compromises the accuracy of long-term quantitative remote sensing applications, making sustained in-orbit radiometric calibration essential for data consistency and climate monitoring [5,6,7,8]. Three approaches are commonly used for in-orbit calibration. These are onboard calibration with internal reference devices, cross-calibration against reference sensors, and vicarious calibration based on ground measurements and radiative transfer modeling [9,10,11]. For high-resolution optical satellites with narrow swaths and limited onboard calibration, simultaneous nadir overpass opportunities are scarce. This scarcity restricts both the applicability and accuracy of cross-calibration [12,13,14,15]. Vicarious calibration is therefore the primary option for such sensors. It yields absolute calibration coefficients that are physically grounded and SI-traceable, and does not depend on the radiometric quality of any reference satellite [16,17].

Conventional vicarious calibration nonetheless requires synchronized in situ measurements during each overpass. Such campaigns impose substantial personnel and logistical demands, which limit the achievable calibration frequency [18]. To overcome this constraint, the Committee on Earth Observation Satellites (CEOS) established the Radiometric Calibration Network (RadCalNet) [19,20,21]. RadCalNet deploys automated instrumentation at globally distributed sites under a unified measurement and processing protocol [21,22]. It provides hyperspectral surface and top-of-atmosphere (TOA) reflectance at a 30 min sampling rate, with concurrent atmospheric parameters, all of which are traceable to SI units [22]. The network is now widely adopted for the in-orbit calibration and validation of optical sensors [21,22].

A key limitation of RadCalNet, however, is that its standard reflectance products are derived under nadir-equivalent viewing geometry [23]. When applied to off-nadir sensors, surface anisotropy, as described by the Bidirectional Reflectance Distribution Function (BRDF), introduces systematic biases. These biases are most pronounced over non-Lambertian targets and at large view zenith angles [24,25,26,27]. The mismatch is particularly relevant for FORMOSAT-5 (FS-5), Taiwan’s first domestically developed high-resolution optical satellite. Its Remote Sensing Instrument (RSI) acquires panchromatic and multispectral imagery for environmental monitoring, disaster assessment, and land-use analysis [28]. However, FS-5 has limited onboard calibration capability and routinely operates at off-nadir viewing angles to extend revisit coverage [28]. Direct use of RadCalNet products in FS-5 calibration, therefore, introduces BRDF-induced biases into the radiative transfer simulation. These biases then propagate to the derived coefficients and influence downstream products. Despite the recognized importance of BRDF effects, the magnitude of this propagation has not been systematically quantified for off-nadir high-resolution missions.

This study addresses this research gap by proposing a BRDF-corrected vicarious calibration framework for the FS-5 RSI. RadCalNet surface reflectance is combined with an empirical BRDF lookup table constructed from in situ multi-angle measurements at the Railroad Valley Playa calibration site. The geometry-matched reflectance is then propagated through 6S radiative transfer simulations to derive the calibration coefficient $K_{0}$. Four FS-5 overpasses spanning a range of viewing geometries are used to evaluate the $K_{0}$ improvement achieved by BRDF correction. To examine the downstream consequences of calibration accuracy, corrected and uncorrected $K_{0}$ values are applied to an FS-5 La Crau scene. The resulting differences in TOA reflectance and the near-infrared reflectance of vegetation (NIRv) are then compared.

NIRv, defined as the product of NDVI and NIR reflectance, is widely used as a proxy for terrestrial gross primary production [29,30,31]. It has been adopted across multiple sensors for long-term ecosystem monitoring, sub-daily carbon flux estimation, and global photosynthesis reconstruction [32,33,34]. The normalized form of NDVI partially compensates for systematic biases of similar magnitude in Red and NIR reflectance. In contrast, NIRv retains direct sensitivity to NIR variations [30]. NIRv is therefore a more appropriate metric for examining how radiometric calibration accuracy propagates into vegetation retrievals.

Accordingly, the specific objectives of this study are threefold: (1) to construct an empirical BRDF lookup table from in situ multi-angle measurements at Railroad Valley Playa and apply it to the conversion of RadCalNet nadir-equivalent surface reflectance to the actual off-nadir viewing geometry of the FS-5 RSI; (2) to quantify the influence of BRDF correction on the retrieval accuracy of the absolute radiometric calibration coefficient $K_{0}$ across all five FS-5 spectral bands; and (3) to evaluate how BRDF-induced calibration differences propagate into downstream products, including TOA reflectance and NIRv, using an independent and spatially heterogeneous FS-5 scene. Through these objectives, this study aims to systematically assess the role of BRDF correction in vicarious calibration and to examine its implications for quantitative applications of high-resolution optical satellite observations. To our knowledge, this is the first study to quantitatively evaluate how BRDF correction propagates from vicarious calibration coefficients to NIRv retrievals for the FORMOSAT-5 mission.

## 2. Datasets and Calibration Site

### 2.1. Dataset

#### 2.1.1. Formosat-5 Data

The FORMOSAT-5 (FS-5) satellite is Taiwan’s first indigenously developed high-resolution optical remote sensing satellite, equipped with a multispectral and panchromatic imaging sensor. It simultaneously acquires panchromatic (PAN) and multispectral (MS) imagery. The PAN band spans the visible to near-infrared spectral range at a 2 m spatial resolution. The MS imagery comprises four bands, Blue (B), Green (G), Red (R), and near-infrared (NIR), at a 4 m spatial resolution. Table 1 summarizes these specifications. This band configuration supports a range of applications including land cover classification, environmental monitoring, and quantitative remote sensing.

**Figure 1:**
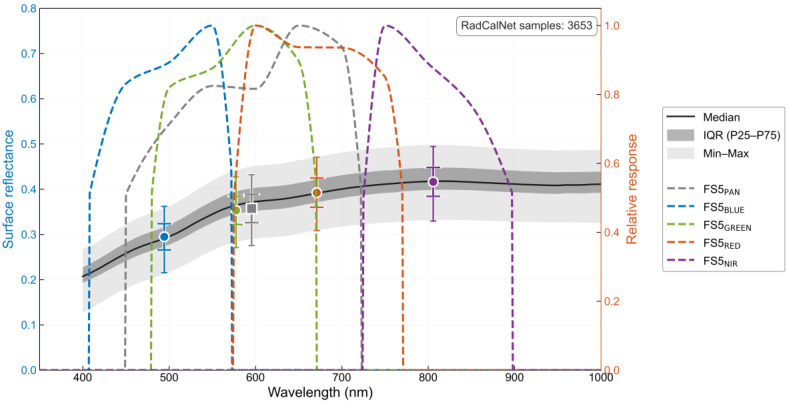
FORMOSAT-5 relative spectral response (SRF; right axis, dashed) overlaid on RadCalNet surface reflectance for Railroad Valley Playa (38.497° N, 115.690° W) from *N* = 3653 daily spectra in 2025. Left axis: Per-wavelength median (solid line), interquartile range (dark band), and min–max envelope (light band). Markers at each band’s centroid show the band-integrated mean ± 1 σ surface reflectance (PAN: square; others: circles); horizontal whiskers indicate min/max.

To facilitate accurate radiometric calibration and quantitative analysis, each FS-5 band is characterized by a corresponding spectral response function (SRF), which describes the sensor’s relative spectral sensitivity as a function of wavelength, as illustrated in Figure 1. The FS-5 RSI SRFs used in this study were provided by the Taiwan Space Agency (TASA). These response functions allow ground-based hyperspectral measurements to be converted into band-equivalent reflectance values for the satellite sensor, and play a critical role in the radiometric calibration process. By integrating the FS-5 band configuration, spatial resolution, and SRFs, a rigorous quantitative calibration framework can be established to estimate the TOA radiometric response from surface reflectance measurements, thereby enhancing the radiometric consistency and long-term stability of the imagery.

#### 2.1.2. RadCalNet

The surface reflectance data used in this study were obtained from RadCalNet, an international ground-based radiometric calibration network. RadCalNet deploys automated measurement systems at globally representative calibration sites to routinely provide standardized hyperspectral surface reflectance data and concurrent atmospheric parameter measurements. All sites adhere to consistent measurement protocols, data processing procedures, and quality control mechanisms, with results traceable to the International System of Units (SI). These standards make RadCalNet a reliable reference for post-launch radiometric calibration and validation of optical imaging sensors [21,35].

RadCalNet provides surface reflectance with a 10 nm spectral sampling interval over 380–2500 nm and a temporal resolution of 30 min. These specifications effectively support the requirements of optical satellite missions with diverse band configurations and overpass times. RadCalNet also provides ancillary atmospheric parameters with each observation record, including aerosol optical depth (AOD), total column ozone (TCO), and total column water vapor (WV). In this study, all required atmospheric input parameters were sourced directly from the RadCalNet dataset. All data are publicly accessible through the official RadCalNet data portal.

### 2.2. Railroad Valley Playa Site

**Figure 2:**
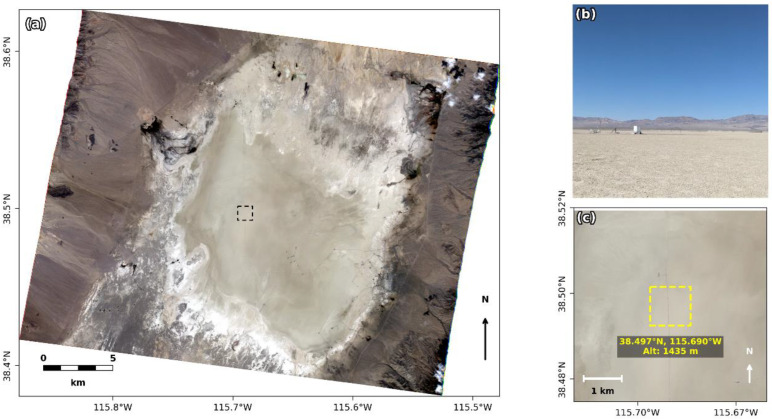
The Railroad Valley Playa calibration site. (**a**) Formosat-5 (FS-5) true-color RGB acquired on 12 September 2025. The dashed rectangle indicates the 1 km^2^ calibration site. (**b**) Ground photograph taken at Railroad Valley Playa, showing the homogeneous bright surface of the dry lakebed and the permanent RadCalNet monitoring station. (**c**) Enlarged FS-5 image of the 1 km^2^ area corresponding to the dashed rectangle in (**a**).

Railroad Valley Playa is one of the core sites of the RadCalNet international radiometric calibration network. It is located in central Nevada, USA (38.497° N, 115.690° W), at an elevation of approximately 1435 m above mean sea level (Figure 2a). The site is among the most widely used standard calibration targets for ground-based vicarious radiometric calibration internationally. The terrain is a dry salt playa with flat topography, high spatial homogeneity, minimal vegetation cover, and limited anthropogenic disturbance (Figure 2b,c). These characteristics produce excellent spectral reflectance stability and long-term temporal consistency across the visible-to-shortwave infrared (VSWIR) spectral range. Due to its large spatial extent and surface homogeneity, Railroad Valley Playa has been extensively used for absolute radiometric calibration, cross-calibration, and long-term stability monitoring of medium- to high-resolution optical satellite sensors [17,36]. At this site, radiometric calibration is typically performed using the reflectance-based method [17,21,37]. The method combines three inputs: ground-based hyperspectral surface reflectance, concurrent atmospheric parameter observations (aerosol optical depth, precipitable water vapor, and ozone column), and radiative transfer modeling. These are used to estimate the top-of-atmosphere radiometric response of the target sensor.

### 2.3. La Crau Validation Site

La Crau, located in southeastern France, is a RadCalNet calibration site. It was used in this study to evaluate the downstream impact of the BRDF-corrected calibration coefficients. In contrast to the bright, spatially homogeneous surface of Railroad Valley Playa, La Crau is a heterogeneous alluvial gravel plain comprising exposed gravel, agricultural areas, and water bodies. This spatial heterogeneity provides a range of contrasting surface types within a single scene, making it well suited for assessing how the $K_{0}$ update propagates into TOA reflectance and vegetation signals across diverse land covers. An independent FS-5 RSI image acquired over La Crau on 21 June 2025 was used for this analysis. It should be noted that the BRDF correction described in Section 3 is applied solely to the vicarious calibration procedure at Railroad Valley Playa; the Railroad-derived BRDF characteristics are not transferred to the La Crau scene. The calibration coefficients, $K_{0}$, obtained from the procedure characterize the FS-5 sensor itself and are, therefore, applicable to any FS-5 observation, including the La Crau scene analyzed in Section 4.4.

## 3. Methodology

### 3.1. Overview of Calibration Framework

**Figure 3:**
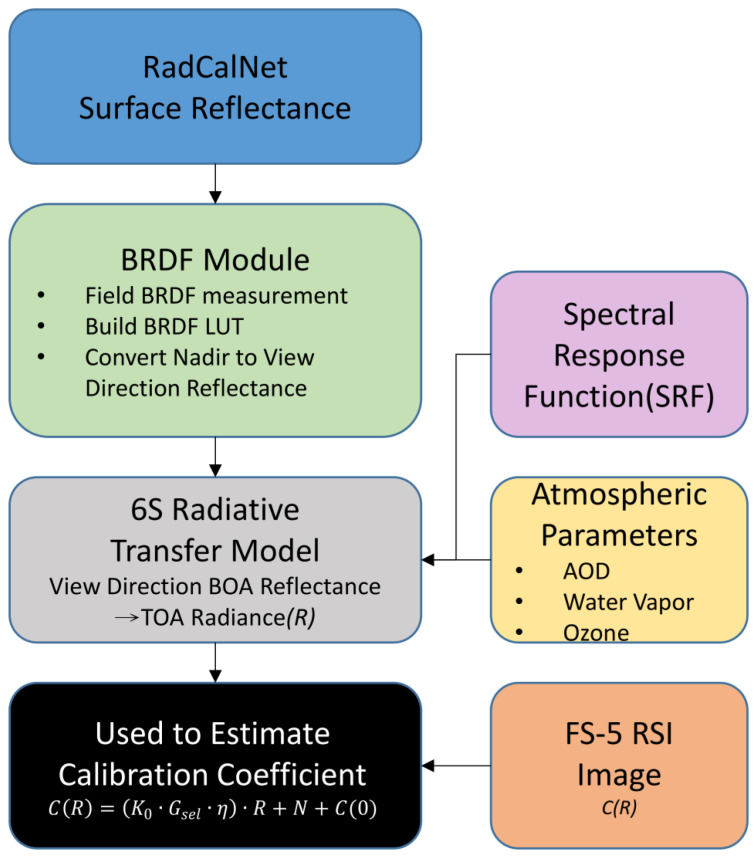
Overview of the vicarious radiometric calibration framework for FS-5 RSI incorporating BRDF correction. The workflow integrates RadCalNet surface reflectance data, in situ BRDF measurements, and FS-5 observation geometry to derive surface reflectance corresponding to the satellite viewing direction. Atmospheric parameters including aerosol optical depth (AOD), water vapor, and ozone are input into the 6S radiative transfer model together with the FS-5 RSI SRFs to simulate band-equivalent TOA radiance. The simulated TOA radiance is subsequently compared with FS-5 image digital numbers (DNs) through the linear radiometric calibration equation to derive the calibration coefficients.

This study establishes a comprehensive vicarious radiometric calibration framework that integrates in situ surface measurements, BRDF correction, and radiative transfer simulation to derive the radiometric response characteristics of FORMOSAT-5 (FS-5) remote sensing imagery. The overall calibration workflow is illustrated in Figure 3. First, a BRDF database was constructed based on in situ BRDF measurements. Ground-based surface spectral reflectance data provided by RadCalNet were then used as the reference surface reflectance input. Since satellite observations are generally acquired at off-nadir geometries, the surface reflectance was adjusted using the BRDF database according to the FS-5 RSI observation geometry, defined by the solar zenith angle, view zenith angle, and relative azimuth angle.

The geometrically corrected surface reflectance was then combined with concurrent atmospheric parameters: aerosol optical depth (AOD), precipitable water vapor, and ozone concentration. These were input into the Second Simulation of the Satellite Signal in the Solar Spectrum (6S) radiative transfer model to compute the corresponding TOA radiance. To ensure spectral consistency, the SRFs of the FS-5 RSI were incorporated into the 6S radiative transfer model as band-specific spectral weighting inputs, enabling the model to directly simulate the band-equivalent TOA radiance corresponding to each FS-5 spectral band.

Finally, the radiometric response relationship between the FS-5 image digital numbers (DNs) and the simulated TOA radiance was established. Based on the radiometric characteristics of the satellite sensor, this relationship can be expressed as follows:

(1) $$C\left(R\right)=\left(K_{0}\cdot G_{sel}\cdot \eta \right)\cdot R+N+C(0)$$

where $C\left(R\right)$ denotes the image digital number, R denotes the TOA radiance, $K_{0}$ denotes the absolute radiometric calibration coefficient, $G_{sel}$ denotes the selected gain setting, η denotes the system amplification factor, N denotes the system noise term, and C(0) denotes the offset. Calibration coefficients ($K_{0}$) were retrieved by comparing the simulated TOA radiance with the corresponding image C(R) values. The impact of BRDF correction on the accuracy of $K_{0}$ is then quantitatively evaluated.

This framework integrates ground-based in situ measurements, observation geometry correction, and a physics-based radiative transfer model, enabling quantitative assessment of BRDF effects on the radiometric calibration of high-resolution optical satellite sensors.

### 3.2. SRF-Weighted Spectral Integration

The hyperspectral surface reflectance data used in this study were obtained from two sources: RadCalNet and in situ ASD field spectroradiometer measurements at Railroad Valley Playa. Both datasets require conversion to FS-5 band-equivalent values via SRF-weighted spectral integration before further analysis. For the radiative transfer simulation in the calibration workflow, the 6S model accepts hyperspectral surface reflectance as input and internally performs SRF-weighted integration to directly output the band-equivalent TOA radiance for each FS-5 spectral band, as expressed in Equation (2):

(2) $$L_{FS5,i}=\frac{\int_{\lambda_{min,i}}^{\lambda_{max,i}}L_{TOA}\left(\lambda \right)\cdot SRF\left(\lambda \right)d\lambda }{\int_{\lambda_{min,i}}^{\lambda_{max,i}}SRF\left(\lambda \right)d\lambda }$$

where $L_{FS5,i}$ denotes the band-equivalent TOA radiance of the i-th FS-5 spectral band, $L_{TOA}\left(\lambda \right)$ denotes the TOA radiance at wavelength λ simulated by the 6S radiative transfer model, $SRF\left(\lambda \right)$ denotes the spectral response function of the corresponding FS-5 band, and $\lambda_{max,i}$ and $\lambda_{min,i}$ denote the upper and lower bounds of the effective wavelength range of that band.

To further facilitate the analysis and visualization of per-band BRDF directional behavior, the in situ hyperspectral surface reflectance measured by ASD was additionally processed through the same SRF-weighted integration scheme, as expressed in Equation (3):

(3) $$\rho_{FS5,i}=\frac{\int_{\lambda_{min,i}}^{\lambda_{max,i}}\rho_{surface}\left(\lambda \right)\cdot SRF\left(\lambda \right)d\lambda }{\int_{\lambda_{min,i}}^{\lambda_{max,i}}SRF\left(\lambda \right)d\lambda }$$

where $\rho_{FS5,i}$ denotes the band-equivalent surface reflectance of the i-th FS-5 spectral band and $\rho_{surface}\left(\lambda \right)$ denotes the surface reflectance at wavelength λ provided by in situ measurement.

These band-equivalent reflectance values are subsequently used to characterize the BRDF directional behavior at the Railroad Valley Playa site and to evaluate the consistency between BRDF-corrected and reference reflectance values across spectral bands.

### 3.3. Viewing Geometry Definition

The solar and satellite observation geometry parameters are critical factors in both BRDF correction and radiometric calibration analysis. In this study, the observation geometry parameters of the FS-5 RSI imagery, including solar zenith angle ($\theta_{s}$), solar azimuth angle ($\varphi_{s}$), view zenith angle ($\theta_{v}$), and view azimuth angle ($\varphi_{v}$), were extracted for subsequent analysis, as summarized in Table 2.

Among these parameters, $\theta_{s}$, $\varphi_{s}$, $\theta_{v}$, and $\varphi_{v}$ were obtained directly from the metadata files accompanying the FS-5 imagery.

Among the FS-5 overpasses concurrent with the on-site multi-angle BRDF measurement campaign at Railroad Valley Playa from 4 to 12 September 2025, four cloud-free scenes (4, 6, 8, and 12 September) with solar zenith angles within the LUT-applicable range ($\theta_{s}$ ≈ 35–38°) were selected for calibration analysis. The 10 September overpass was excluded due to cloud contamination identified through on-site weather observation. The view zenith angles of the four selected scenes range from approximately 27.2° to 27.9° (Table 2), which is well within the LUT’s valid angular coverage ($\theta_{v}$ ≤ 60°); no scenes were excluded by the $\theta_{v}$ threshold criterion in the present analysis.

### 3.4. Empirical BRDF Lookup Table Construction from In Situ Measurements

To characterize the directional reflectance properties of the calibration site surface, an empirical Bidirectional Reflectance Distribution Function (BRDF) lookup table (LUT) was constructed through in situ multi-angle surface reflectance measurements. Ground-based hyperspectral surface reflectance measurements were conducted using an ASD FieldSpec spectroradiometer (ASD Inc., Boulder, CO, USA) (spectral range: 350–2500 nm) [38,39]. Under each observation geometry condition, the radiance of the target surface and a Spectralon reference panel were measured alternately, and the surface spectral reflectance was calculated according to the following equation:

(4) $$\rho \left(\lambda \right)=\frac{L_{target}(\lambda )}{L_{panel}(\lambda )}\cdot \rho_{panel}$$

where $L_{target}$ and $L_{panel}$ denote the radiance measured from the target surface and the reference panel, respectively, and $\rho_{panel}$ denotes the reflectance of the reference panel. In this study, a Spectralon reference panel with a nominal reflectance of $\rho_{panel}=1$ was used for all field measurements.

To acquire surface reflectance under varying observation geometries, multi-angle measurements were conducted across a range of view zenith angles ($\theta_{v}$) and azimuth angles ($\varphi_{v}$). The measurement geometry was configured with $\theta_{v}$ ranging from 0° to 60° at 20° intervals; azimuth angles ranged from 0° to 360° at 45° intervals measured clockwise from the north. To minimize the influence of instantaneous noise and outliers, measurements were repeated three times at each angular node. The median of the three replicates was adopted as the representative reflectance, thereby enhancing the robustness of the empirical BRDF LUT. The BRDF LUT can be expressed as follows:

(5) $$\rho_{BRDF}=f(\theta_{v},\varphi )$$

$$\varphi =\varphi_{v}-\varphi_{s}$$

where $\theta_{v}$ denotes the satellite view zenith angle, φ denotes the relative azimuth angle, $\varphi_{s}$ denotes the solar azimuth angle, and $\varphi_{v}$ denotes the satellite view azimuth angle.

Since the in situ measurements were acquired at discrete angular nodes, linear interpolation was used to estimate reflectance values for satellite observation geometries that do not coincide exactly with the measured nodes. The interpolation procedure simultaneously accounts for both satellite view zenith angle ($\theta_{v}$) and the relative azimuth angle (φ) between the solar and satellite viewing directions.

The BRDF LUT employed in this study was constructed from in situ measurements conducted at Railroad Valley Playa on 12 September 2025 between 17:58 and 19:00 UTC. During this measurement period, the solar zenith angle ranged from 41.5° to 34.9°, and the solar azimuth angle increased from 140.2° to 163.8°. This indicates a limited but non-negligible variation in solar geometry. As the BRDF database was established from a single measurement session, its applicable solar geometry conditions correspond to an $\theta_{s}$ range of approximately 35–42°. Consequently, BRDF geometric normalization in this study was applied exclusively to satellite images with $\theta_{s}$ values falling within this range, thereby avoiding extrapolation of the empirical LUT to substantially different solar geometry conditions. Similarly, the LUT is strictly applicable within the measured $\theta_{v}$ range of 0–60° to prevent instability caused by angular extrapolation; satellite observations with $\theta_{v}$ exceeding this range are excluded from BRDF correction.

The LUT-based approach enables geometric normalization using empirically measured surface directional reflectance. As the LUT represents the directional reflectance under the specific solar geometry of a single measurement session, its applicability is constrained to the $\theta_{v}$ and $\theta_{s}$ ranges described above.

### 3.5. BRDF Correction

RadCalNet provides nadir-equivalent surface reflectance ($\theta_{v}$ = 0°), which introduces a geometric discrepancy relative to the off-nadir observation conditions of the FS-5 RSI. Given that the surface reflectance of Railroad Valley Playa exhibits pronounced directional characteristics, any failure to account for this angular difference would introduce systematic biases into the estimation of radiometric calibration coefficients. Therefore, to ensure geometric consistency between the RadCalNet-derived surface reflectance and the actual observation geometry of the FS-5 RSI, a BRDF-based directional correction was applied prior to vicarious calibration.

To address this geometric discrepancy, the empirical BRDF LUT constructed in this study was employed to convert the RadCalNet nadir-equivalent reflectance to the actual observation geometry of the FS-5 RSI. The conversion is defined as follows:

(6) $$\rho_{BRDF}\left(\lambda ,\theta_{v},\varphi \right)=\rho_{RadCalNet}\left(\lambda \right)\cdot \frac{\rho_{LUT}(\lambda ,\theta_{v},\varphi )}{\rho_{LUT}(\lambda ,0,0)}$$

where $\rho_{BRDF}\left(\lambda ,\theta_{v},\varphi \right)$ denotes the geometrically corrected surface reflectance at the FS-5 observation geometry; $\rho_{RadCalNet}$ denotes the nadir-equivalent surface reflectance provided by RadCalNet; $\rho_{LUT}(\lambda ,\theta_{v},\varphi )$ denotes the LUT-interpolated reflectance at the target observation geometry; and $\rho_{LUT}(\lambda ,0,0)$ denotes the nadir-equivalent reflectance from the LUT used as the normalization reference. During this conversion, the RadCalNet surface reflectance is adjusted so it remains consistent with the actual observation geometry of the FS-5 RSI, thereby ensuring that both datasets are compared and calibrated under equivalent directional reflectance conditions.

### 3.6. TOA Reflectance and NIRv Computation

This study subsequently applied the updated radiometric calibration coefficients to convert the satellite image digital numbers (DNs) into TOA radiance. To eliminate the influence of solar irradiance, the Earth–Sun distance and solar zenith angle were taken into account in the radiometric measurements. The TOA radiance was further converted to TOA reflectance using the following equation:

(7) $$\rho =\frac{\pi \cdot R\cdot d^{2}}{E_{sun}\cdot \cos\theta_{s}}$$

where R denotes the TOA radiance, d denotes the Earth–Sun distance correction factor, $E_{sun}$ denotes the band-integrated solar exo-atmospheric spectral irradiance (W m^−2^ μm^−1^) for each FS-5 spectral band, computed by integrating the hyperspectral solar irradiance [40] weighted by the corresponding FS-5 SRF, and $\theta_{s}$ denotes the solar zenith angle.

TOA reflectance was subsequently used to compute NIRv without applying atmospheric correction to focus the evaluation on the direct influence of $K_{0}$ updates on NIRv retrieval. NIRv was calculated using the following equations:

(8) $$NDVI=\frac{\rho_{NIR}-\rho_{Red}}{\rho_{NIR}+\rho_{Red}}$$

(9) $$NIRv=\rho_{NIR}\cdot NDVI$$

Two sets of TOA reflectance and NIRv images were independently computed using $K_{0}$ derived without BRDF correction and $K_{0}$ derived with BRDF correction, respectively. The difference between the two was quantified using the relative difference, defined as follows:

(10) $$Relative\;difference=\frac{X_{BRDF}-X_{woBRDF}}{\bar{X_{woBRDF}}}\cdot 100\%$$

where X represents the quantity of interest (i.e., either band-specific TOA reflectance or NIRv), $X_{BRDF}$ denotes the per-pixel value computed using BRDF-corrected $K_{0}$, and $\bar{x_{woBRDF}}$ denotes the scene-mean value computed using $K_{0}$ without BRDF correction. The scene-mean is used in the denominator, rather than the per-pixel value, to avoid singularities where the uncorrected quantity approaches zero (e.g., over water bodies or sparsely vegetated areas in the case of NIRv). This yields a well-conditioned and spatially interpretable measure of the calibration-induced relative change. Positive values indicate a relative increase in X following BRDF correction, while negative values indicate a relative decrease. The spatial distribution of the relative difference, together with scene-level statistical comparisons including pixel mean and standard deviation, was used to evaluate the spatial extent and characteristics of the $K_{0}$ update and its impact on TOA reflectance and NIRv retrieval.

In the subsequent TOA reflectance and NIRv analyses, only the four FS-5 multispectral bands (Blue, Green, Red, and NIR) were considered, as the panchromatic band is not employed in the vegetation index formulations examined in this study.

All data processing was performed using MATLAB (R2022a and R2026a) and Python 3.14 (with NumPy 2.4.4, SciPy 1.17.1, Rasterio 1.5.0, Pandas 3.0.2, Matplotlib 3.10.8, and Pillow 12.2.0).

## 4. Results and Discussion

### 4.1. BRDF at the Railroad Site

**Figure 4:**
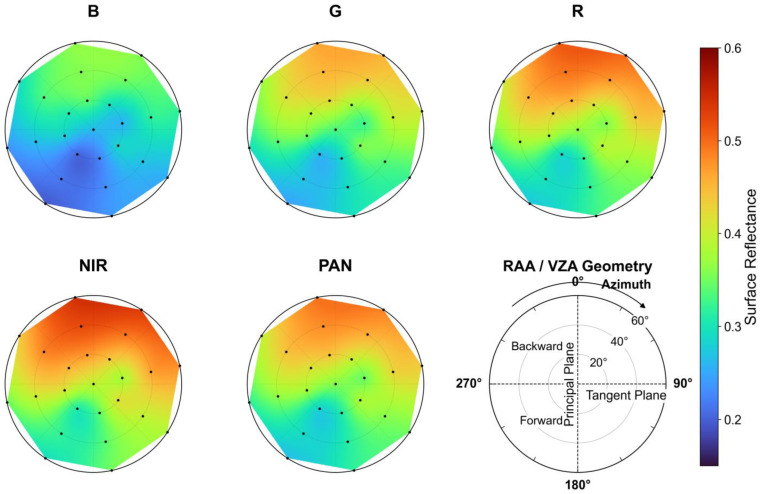
Angular distribution of surface reflectance for the five FS-5 spectral bands (B, G, R, NIR, and PAN) at Railroad Valley Playa, derived from in situ multi-angle measurements with an ASD FieldSpec spectroradiometer. The radial axis represents the view zenith angle ($\theta_{v}$), with concentric circles at $\theta_{v}$ = 20°, 40°, 60°; the angular axis represents the relative azimuth angle φ, with φ = 0° (top) corresponding to the backscatter/hot-spot direction and φ = 180° (bottom) corresponding to the forward-scatter direction. All panels share a common colour scale (0.15–0.60) to enable cross-band comparison; black dots represent locations where samples were measured. The lower-right panel illustrates the φ–$\theta_{v}$ geometry convention and principal-plane orientations adopted in this study.

Figure 4 presents the angular distribution of the FS-5 band-equivalent surface reflectance derived from in situ multi-angle measurements at Railroad Valley Playa (Section 3.4). All five spectral bands (B, G, R, NIR, and PAN) exhibit consistent and smooth directional reflectance distributions. Reflectance values are highest in the backscatter direction (φ ≈ 0°) and systematically decrease toward the forward scatter direction (φ ≈ 180°). This trend is consistent across all bands, indicating pronounced non-Lambertian reflectance characteristics of the calibration site surface, which is in agreement with the known scattering properties of dry playa surfaces [25].

The surface of Railroad Valley Playa exhibits partial micro-relief and macroscopic roughness, causing its reflectance behavior to display an asymmetric angular distribution in response to varying solar and viewing geometries. The shadowing effect induced by surface roughness is the primary mechanism behind this asymmetric BRDF pattern. In the forward scattering direction (φ ≈ 180°), micro-relief features cast shadows that systematically reduce the overall reflected signal. In the backscatter direction (φ ≈ 0°), a greater proportion of the surface remains illuminated, and shadowing effects are minimal. This directional behavior is consistent with findings reported for Railroad Valley Playa and similar dry playa surfaces [25,41]. This is further confirmed by the directional reflectance patterns observed in this study.

### 4.2. Surface Reflectance with BRDF Correction

Table 3 compares three sets of FS-5 band-equivalent surface reflectance—in situ ASD measurements acquired under the actual FS-5 observation geometry, RadCalNet nadir-equivalent reflectance, and BRDF-corrected reflectance (converted to the FS-5 geometry via the empirical LUT)—with the ASD measurements taken as the reference.

As shown in Table 3, directly applying the nadir reflectance yields systematic negative biases relative to the ASD measurements across all spectral bands. The relative errors range from approximately −13% to −15%. This indicates that neglecting surface directional effects leads to a systematic underestimation of reflectance under actual observation conditions. Following BRDF correction, the relative errors are substantially reduced to approximately −1% to −4% across all bands. Consistent improvement is observed in both the visible and near-infrared spectral ranges. This demonstrates the good spectral consistency of the BRDF correction. These results indicate that incorporating BRDF correction effectively reduces systematic biases introduced by differences in observation geometry. This has critical implications for improving the accuracy of the radiometric calibration coefficient $K_{0}$ and the reliability of quantitative satellite data.

### 4.3. Vicarious Calibration Results

This study further evaluates how different surface reflectance sources affect the estimation of $K_{0}$. Using the calibration results derived from ASD in situ measurements as the reference baseline, we compare the calibration accuracy between uncorrected RadCalNet nadir reflectance and BRDF-corrected reflectance.

**Figure 5:**
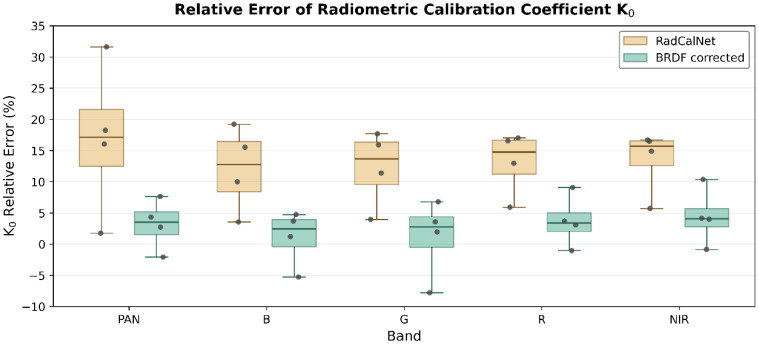
Impact of BRDF correction on the relative error of the radiometric calibration coefficient $K_{0}$ for each FS-5 spectral band (PAN, B, G, R, and NIR). Relative errors were evaluated against ASD in situ measurements as the reference baseline. Boxplots compare $K_{0}$ relative errors derived from RadCalNet nadir surface reflectance without BRDF correction (Orange) and with BRDF correction (Green). The results demonstrate that incorporating BRDF correction substantially reduces the systematic bias between RadCalNet-based calibration coefficients and ASD in situ reference values across all spectral bands. Each box represents four FS-5 acquisitions over Railroad Valley Playa (Table 2), with individual measurements overlaid as black dots. The corresponding per-acquisition $K_{0}$ values are reported in Table 3.

As shown in Figure 5, when RadCalNet nadir reflectance was directly applied for calibration, the median $K_{0}$ relative errors across all spectral bands ranged from approximately 13% to 17%. The overall distribution spanned approximately 8% to 33%. The PAN band exhibited the widest error distribution, with the upper whisker reaching approximately 33%, indicating that the systematic bias introduced by off-nadir observation geometry is most pronounced in this band. Following BRDF correction, the median $K_{0}$ relative errors were substantially reduced, with consistent improvement observed across all spectral bands. This demonstrates the good spectral consistency of BRDF correction and its effectiveness in reducing systematic biases resulting from differences in observed geometry.

To assess the robustness of this improvement, and because each band-specific result is based on four FS-5 overpasses (*n* = 4), formal significance testing was not pursued. Instead, robustness was evaluated using the consistency of error reduction across all bands and acquisitions. The median $K_{0}$ relative error decreased from 13–17% to 1–4%, while the overall error range decreased from approximately 8–33% to approximately −5% to 10%. The fact that this improvement was observed consistently across all five bands and four acquisitions suggests that BRDF correction produced a repeatable reduction in calibration error for the set of scenes analyzed in this study.

These findings are consistent with the conclusions of previous studies. Han et al. [42] used annual official calibration coefficients (OCCs) from the Dunhuang site as the validation reference. They reported that $K_{0}$ relative errors for the GF-6 WFV sensor reached up to 13.19% without BRDF correction and were reduced to within 8.48% after correction. Pan et al. [27] used pre-launch laboratory calibration coefficients as the reference baseline. They demonstrated that the mean relative difference in calibration coefficients for the CBERS-04 WFI sensor decreased from 5.42% to 1.74% following BRDF correction. Both studies confirm that BRDF correction effectively improves radiometric calibration accuracy, consistent with the improvement trend observed in this study.

Nevertheless, discrepancies in error magnitude are evident among studies, which can be attributed to the following factors. First, the evaluation baselines differ. This study uses ASD in situ multi-angle measurements as the reference, while Han et al. [42] adopt OCCs derived from field observations at the Dunhuang site, and Pan et al. [27] employ pre-launch laboratory coefficients. The different reference baselines and their associated uncertainties make direct comparison of the absolute error magnitudes across studies difficult. Second, although the $\theta_{v}$ ranges of FS-5 RSI ($\theta_{v}$ ≈ 27–28°), GF-6 WFV ($\theta_{v}$ ≈ 6–28°), and CBERS-04 WFI ($\theta_{v}$ ≈ 3–28°) are comparable, the sensors differ in SRFs, swath width design, and image acquisition conditions. These differences may contribute to variations in the effectiveness of BRDF correction.

To contextualize the BRDF-derived calibration improvement to other potential sources of error, we further quantified the sensitivity of $K_{0}$ to uncertainties in azimuth angle in the FS-5 metadata through perturbation analysis around the baseline acquisition geometry (Supplementary Material, Section S1). Independent and joint perturbations of the solar azimuth (φ_s_) and view azimuth (φ_v_) were applied with Δφ_s_, Δφ_v_ ∈ {±1°, ±2°, ±3°}, and the full BRDF–6S–$K_{0}$ pipeline was re-executed for each of the 15 cases. The results show that $K_{0}$ depends only on the relative azimuth ΔRAA = φ_s_ − φ_v_, with a near-linear response of approximately 0.21–0.24% per degree across the five FS-5 bands. In the worst case (|ΔRAA| = 3°), the corresponding |Δ$K_{0}$| remains below 0.75% in the most sensitive band (Blue). This magnitude is substantially smaller than the 9–16 percentage-point systematic $K_{0}$ bias addressed by the BRDF correction itself (Table 3), indicating that azimuth metadata uncertainty is a secondary error source compared with the BRDF effect in FS-5 $K_{0}$ retrieval at Railroad Valley Playa.

A further methodological consideration is the use of an empirical lookup table (LUT) or a parametric kernel-driven BRDF model such as the RossThick–LiSparse-Reciprocal (RTLSR) or the Rahman–Pinty–Verstraete (RPV) formulation. The LUT representation adopted here directly preserves the measured anisotropy without imposing a specific functional form, thereby avoiding potential model-fitting residuals that arise when the assumed kernels do not fully capture the surface’s scattering behavior. Linear interpolation between the discrete angular nodes was selected for the same reason: it introduces no parametric assumption and is adequate within the densely sampled angular range relevant to the FS-5 viewing geometries ($\theta_{v}$ ≈ 27°, with LUT nodes at 0°, 20°, 40°, and 60° in $\theta_{v}$ and at 45° intervals for the azimuth angle). Kernel-driven models, by contrast, provide a continuous, analytically extrapolable BRDF representation across the full angular hemisphere and would be preferable when correction is required for geometries far from the measured range, or when globally smooth parameterization is needed for operational pipelines. For the present application, where the relevant FS-5 viewing geometry falls well within the LUT’s measured angular coverage, the empirical LUT provides a direct and assumption-free correction; extending the framework to wider geometric ranges or to multi-temporal operational use would warrant complementary kernel-driven modeling.

Beyond the methodological considerations discussed above, the operational feasibility of the proposed framework warrants brief comment. As currently formulated, the framework is best suited for post hoc, analysis-ready product generation rather than near-real-time calibration. This is because the framework relies on a pre-constructed empirical LUT derived from a dedicated field campaign and the delivery of RadCalNet site reflectance data with non-trivial latency. For a continuous operational calibration service, two implementation pathways are conceivable. The first assumes that the BRDF anisotropy of a well-established pseudo-invariant calibration site such as Railroad Valley Playa remains sufficiently invariant between validation campaigns. Under this assumption, the existing LUT can be applied operationally until the next scheduled field measurement, reducing dependence on repeated field campaigns. Periodic verification with independent multi-angle observations would still be advisable to detect any long-term drift in the site’s directional reflectance properties. The second pathway adopts kernel-driven BRDF parameterization that can be updated using routinely available multi-angle satellite observations, enabling continuous adjustment without dedicated field campaigns. A hybrid strategy combining the two approaches may offer an optimal balance. Anchoring the calibration on a field-derived empirical LUT while using a kernel-driven model for temporal interpolation between campaigns would preserve the site-specific accuracy of empirical measurements while providing the temporal flexibility required by operational data pipelines.

The BRDF LUT in this study was effective within its measured $\theta_{s}$ range of 35–42° and $\theta_{v}$ range of 0–60°. Its applicability under different seasonal solar geometries nonetheless warrants further discussion. The macroscopic-roughness shadowing model of Hapke [41] indicates that surface directional anisotropy depends on the illumination and viewing geometry. Such geometries include the lower $\theta_{s}$ encountered in summer and the higher $\theta_{s}$ encountered in winter. Under these conditions, the directional anisotropy of Railroad Valley Playa, and hence the correction factor, may differ from that captured by the current LUT. The angular response of the surface may therefore change. These effects were not measured in the present campaign and cannot be quantified from the current dataset. The actual behavior could therefore deviate from the directional reflectance observed in this study. Applications outside the measured $\theta_{s}$ range would therefore require further validation. They would also likely benefit from seasonal LUT updates acquired under different illumination conditions. In the long term, such updates could be complemented by kernel-driven BRDF parameterizations to provide continuous angular and seasonal interpolation between field campaigns.

A related consideration is the transferability of the proposed framework to darker or vegetated surfaces. It is important to distinguish the correction methodology from the site-specific empirical LUT. The methodology itself consists of deriving a directional reflectance description from in situ multi-angle measurements, normalizing the reference reflectance to the satellite viewing geometry, and propagating it through 6S. As such, it is surface-independent and not restricted to bright targets. The LUT constructed in this study, however, characterizes the specific anisotropy of the Railroad Valley Playa surface and cannot be transferred directly to surfaces with markedly different scattering behavior. This constraint is consistent with the design of vicarious calibration. Bright, flat, and spatially homogeneous pseudo-invariant sites are deliberately selected because their stable and well-characterized reflectance minimizes confounding factors, and the absolute coefficients $K_{0}$ derived at such sites characterize the sensor itself and are subsequently applicable to any scene.

Darker and vegetated surfaces typically exhibit stronger and more complex directional anisotropy arising from volumetric scattering, canopy structure, and hot-spot effects. Consequently, neglecting BRDF effects over such surfaces is expected to induce larger biases than those observed over pseudo-invariant desert calibration sites. This expectation is qualitatively consistent with the pronounced NIRv differences observed over the vegetated portions of the La Crau scene (Section 4.4). However, the present study did not directly quantify BRDF-induced calibration errors for vegetated targets. Characterizing these surfaces requires denser multi-angle sampling and would likely benefit from kernel-driven BRDF models that represent volumetric scattering, rather than a purely empirical LUT.

It is also instructive to contextualize the present approach to radiometric calibration practices for other optical missions such as Landsat 8/9 and Sentinel-2. For these well-characterized sensors, RadCalNet and pseudo-invariant calibration sites are widely used for radiometric validation, monitoring, and cross-comparison of operational Level-1 products [22,23]. Previous studies have demonstrated that BRDF effects can introduce non-negligible biases in cross-sensor and vicarious calibration if differences in observation geometry are not adequately accounted for [14,23,42].

To mitigate these effects, Landsat and Sentinel-2 studies commonly adopt one or more of the following approaches: (1) restricting analyses to near-nadir observations where BRDF effects are minimized; (2) applying kernel-driven BRDF normalization methods, including the RossThick–LiSparse-Reciprocal (RTLSR) formulation used in nadir BRDF-adjusted reflectance (NBAR) processing, to reduce directional reflectance effects [43,44]; and (3) using spectral band adjustment factors (SBAFs) together with pseudo-invariant calibration sites such as Libya-4 and Railroad Valley Playa to reduce angular and spectral inconsistencies [14,23].

The framework proposed here addresses a different operational scenario. FORMOSAT-5 has a more limited onboard radiometric calibration capability than Landsat 8/9 or Sentinel-2 and routinely acquires imagery at off-nadir viewing geometries (~27–28° in the present study). Consequently, directional reflectance effects cannot be effectively minimized through selecting geometry alone and must instead be explicitly incorporated into the absolute vicarious calibration process. Although Landsat 8/9 and Sentinel-2 also employ vicarious calibration and radiometric validation over sites such as Railroad Valley Playa and Libya-4 [16,23], these activities are performed within mature calibration frameworks anchored by onboard calibration subsystems and long-term monitoring programs, so that BRDF treatments are most commonly applied to improve cross-comparison consistency and surface reflectance normalization. In the present study, by contrast, BRDF correction directly influences the retrieval of the vicariously derived calibration coefficient $K_{0}$, thereby affecting the calibration solution and its subsequent validation.

In summary, incorporating BRDF effects into the vicarious calibration workflow can substantially reduce systematic errors in $K_{0}$ estimation. This is particularly critical for high-resolution optical satellites such as FS-5 that lack a comprehensive onboard calibration system. The BRDF LUT constructed in this study was based on a single measurement session and may introduce interpolation uncertainties for angular conditions not densely sampled. The LUT nodes were acquired in close temporal succession within this session (Section 3.4), so the solar geometry varied only slightly across nodes. As the FS-5 overpass geometries analyzed in this study fall within the same narrow $\theta_{s}$ range (35.5–38.0°), this intra-session solar variation is implicitly accounted for by matching LUT nodes to FS-5 observations of comparable $\theta_{s}$. A more rigorous separation of view-angle and solar-angle effects would require a denser multi-session measurement campaign. Future integration of goniometer-based full-hemisphere multi-angle measurements would further reduce uncertainties associated with reflectance conversion and radiometric calibration (see Supplementary Material, Section S2).

### 4.4. Impact of BRDF-Corrected Radiometric Calibration on TOA Reflectance and Vegetation Signal

Using the calibration coefficients derived in Section 4.3, we examine how the BRDF-induced $K_{0}$ update propagates into TOA reflectance and near-infrared reflectance of vegetation (NIRv) for the FS-5 La Crau acquisition described in Section 2.3.

**Figure 6:**
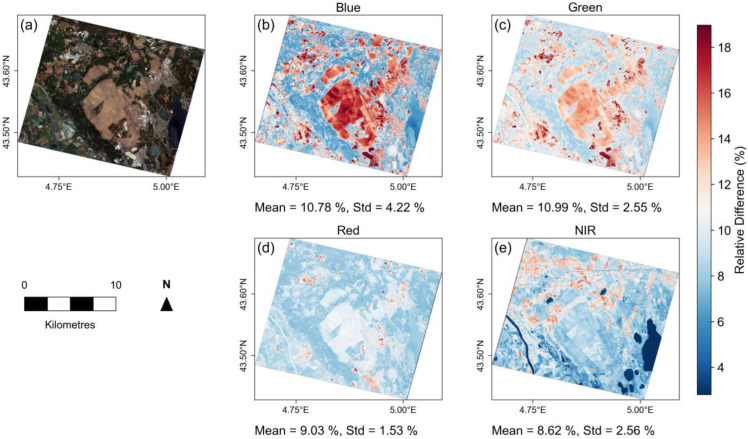
Spatial distribution of relative differences in TOA reflectance between BRDF-corrected and RadCalNet-derived products for each spectral band of Formosat-5 (La Crau, France, 21 June 2025). (**a**) Formosat-5 true-color RGB composite image. (**b**–**e**) Spatial distribution of the relative TOA reflectance difference for each spectral band. The color scale progresses from Blue to Red, indicating an increasing relative difference.

As shown in Figure 6b–e, TOA reflectance was computed separately using $K_{0}$ derived with and without BRDF correction for each spectral band, and the results are presented as relative difference maps. The scene-mean relative differences ranged from 8.62% (NIR) to 10.99% (Green), with standard deviations ranging from 1.53% (Red) to 4.22% (Blue). The spatial distribution of differences exhibits pronounced land-cover-dependent characteristics. Agricultural fields and exposed gravel surfaces with high reflectance have relatively larger differences, whereas low-reflectance areas such as water bodies show smaller differences. This indicates that the $K_{0}$ update has a heterogeneous impact across different surface types. The Blue band exhibited the highest standard deviation (4.22%), demonstrating that the greatest spatial variability in TOA reflectance sensitivity to $K_{0}$ correction took place in this band. These results demonstrate that $K_{0}$ changes introduced by BRDF correction systematically affect TOA reflectance across all spectral bands, with the magnitude of impact varying regarding surface characteristics.

**Figure 7:**
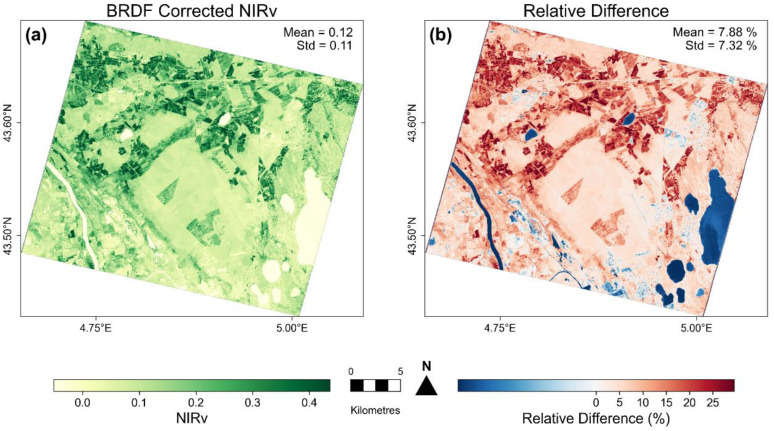
Impact of $K_{0}$ update on near-infrared reflectance of vegetation (NIRv) derived from FS-5 RSI imagery. (**a**) NIRv image computed using BRDF-corrected $K_{0}$ (mean = 0.12, std = 0.11), reflecting the spatial distribution of vegetation density across the scene. (**b**) Relative difference map between NIRv derived from $K_{0}$ without and with BRDF correction.

The impact of $K_{0}$ updates on vegetation parameter retrieval was further evaluated using NIRv, as shown in Figure 7. The NIRv image derived from BRDF-corrected $K_{0}$ (Figure 7a) yielded a scene-mean value of 0.12 with a standard deviation of 0.11, reflecting the high spatial heterogeneity of vegetation cover within the scene analyzed. Figure 7b presents the relative difference in NIRv resulting from the $K_{0}$ update between the BRDF-corrected and uncorrected cases, with a scene-mean relative difference of 7.88% and a standard deviation of 7.32%. The differences are most pronounced over densely vegetated agricultural areas, where pixel-level differences exceed 20% in some locations. By contrast, negative differences are observed over water bodies. This pattern reflects the non-linear response of NIRv to changes in $K_{0}$ under low-vegetation conditions.

These results demonstrate that the accuracy of the radiometric calibration coefficient $K_{0}$ has a direct and non-negligible impact on quantitative remote sensing applications. Even over a spatially heterogeneous scene such as La Crau, the BRDF-corrected $K_{0}$ consistently improves both TOA reflectance and NIRv across all spectral bands.

Badgley et al. [29] reported an approximately linear relationship between NIRv and GPP. Given this relationship, a scene-mean of 7.88% NIRv bias translates directly into a comparable bias in GPP estimates derived from FS-5 vegetation products. The magnitude of this bias is comparable to typical satellite-based GPP uncertainty targets, underscoring the operational importance of BRDF-corrected calibration for carbon cycle applications.

## 5. Conclusions

This study investigated the impact of surface reflectance anisotropy on vicarious radiometric calibration of the FORMOSAT-5 RSI using RadCalNet measurements, in situ ASD multi-angle observations, and radiative transfer modeling. The results demonstrate that the surface reflectance of Railroad Valley Playa exhibits pronounced non-Lambertian behavior that is strongly influenced by viewing geometry. The resulting directional reflectance variations introduce geometry-dependent biases that propagate directly into radiometric calibration if left uncorrected.

An empirical BRDF LUT was used to correct the nadir-equivalent RadCalNet reflectance to the actual FS-5 observation geometry. This reduced the median relative error of the calibration coefficient $K_{0}$ from approximately 13–17% to 1–4% across all spectral bands. These results demonstrate the critical role of BRDF correction in mitigating geometry-induced calibration uncertainties.

The impact of $K_{0}$ updates on quantitative remote sensing retrievals was further assessed using the near-infrared reflectance of vegetation (NIRv). Following the application of BRDF-corrected $K_{0}$, NIRv values exhibited systematic differences relative to those derived from uncorrected coefficients. A scene-mean relative difference of approximately 7.88% and localized differences exceeding 20% over densely vegetated areas were obtained. These results confirm that radiometric calibration accuracy has a direct and non-negligible effect on vegetation parameter retrieval, with implications for land surface monitoring and long-term time-series analysis.

The findings of this study are particularly relevant for high-resolution optical satellite missions such as FS-5, which lack comprehensive onboard calibration systems and therefore rely on ground-based vicarious calibration for post-launch radiometric characterization. The proposed BRDF-based calibration framework provides a physically consistent and operationally feasible approach for improving radiometric accuracy, with direct benefits for quantitative applications including surface reflectance retrieval, vegetation monitoring, and long-term environmental and climate-related observations. More broadly, the framework demonstrates a transferable approach for vicarious calibration of any optical satellite mission lacking comprehensive onboard calibration systems.

The present study was limited to a single calibration site (Railroad Valley Playa) and imagery acquired in September 2025 because the empirical BRDF LUT was constructed from a dedicated in situ multi-angle measurement campaign conducted during that period. Only FS-5 overpasses acquired within the corresponding solar geometry range were analyzed to avoid angular extrapolation beyond the measured BRDF domain. While this limitation is not expected to directly affect the applicability of the derived sensor calibration coefficients, additional campaigns at other sites and in other seasons are needed to evaluate the broader applicability of the proposed framework across different landcover types and seasonal illumination conditions. Furthermore, although the present study demonstrates internally consistent improvements relative to ASD in situ measurements, an independent validation against operational calibration coefficients, cross-calibrated reference sensors, or long-term monitoring datasets was beyond the scope of this work and remains an important direction for future investigation.

Future work will focus on incorporating systematic multi-angle goniometer measurements across multiple calibration sites and seasonal solar geometries to characterize surface reflectance anisotropy for a broader range of solar and viewing geometries. This will reduce interpolation uncertainties in the BRDF LUT and further improve the accuracy of vicarious radiometric calibration.

## Figures and Tables

**Table 1:** Main characteristics of the FORMOSAT-5 Remote Sensing Instrument (RSI). Band 1 is panchromatic, while Bands 2–5 are multispectral. All bands share the same swath width of 24 km.

Band Number	Spectrum Range (nm)	Spatial Resolution (m)	Swath (km)
1	450–722.5	2	24
2	407.5–572.5	4	24
3	480–670	4	24
4	575–770	4	24
5	725–897.5	4	24

**Table 2:** Summary of solar and viewing geometry for FS-5 scenes used in this study. All angular quantities are expressed in degrees.

Scene ID	Date (UTC)	Time (UTC)	$\theta_{s}$ (°)	$\varphi_{s}$ (°)	$\theta_{v}$ (°)	$\varphi_{v}$ (°)
FS5_01	4 September 2025	18:33	35.46	149.16	27.17	101.25
FS5_02	6 September 2025	18:33	36.10	149.84	27.39	101.12
FS5_03	8 September 2025	18:32	36.74	150.56	27.55	101.99
FS5_04	12 September 2025	18:32	38.04	151.93	27.92	101.69

**Table 3:** Comparison of surface reflectance across spectral bands for the four FS-5 overpasses. ASD denotes in situ measurements at the FS-5 observation geometry; Nadir denotes nadir-equivalent reflectance provided by RadCalNet without BRDF correction; BRDF-corrected framework denotes RadCalNet reflectance adjusted to the FS-5 observation geometry through BRDF correction. Surface reflectance (SR) and relative errors (RE, %) are presented; relative errors are computed against the ASD reference.

Date	Metric	PAN			B			G			R			NIR		
		ASD	Nadir	BRDF-Corr.	ASD	Nadir	BRDF-Corr.	ASD	Nadir	BRDF-Corr.	ASD	Nadir	BRDF-Corr.	ASD	Nadir	BRDF-Corr.
4 September 2025	SR	0.40	0.34	0.39	0.34	0.29	0.33	0.40	0.34	0.39	0.44	0.37	0.42	0.46	0.40	0.45
	RE (%)		−15.42	−4.23		−16.67	−4.39		−15.25	−3.50		−14.81	−3.42		−14.66	−4.09
6 September 2025	SR	0.38	0.34	0.39	0.31	0.29	0.33	0.38	0.34	0.39	0.42	0.38	0.43	0.44	0.40	0.45
	RE (%)		−9.76	2.11		−7.72	5.47		−9.55	2.92		−10.69	0.95		−10.16	0.90
8 September 2025	SR	0.42	0.35	0.39	0.35	0.29	0.33	0.42	0.35	0.39	0.47	0.38	0.43	0.50	0.40	0.45
	RE (%)		−18.01	−7.11		−15.61	−3.47		−18.05	−6.41		−19.15	−8.30		−19.40	−9.40
12 September 2025	SR	0.40	0.35	0.39	0.34	0.29	0.33	0.40	0.35	0.40	0.45	0.38	0.44	0.47	0.40	0.46
	RE (%)		−13.86	−2.48		−13.57	−3.45		−13.68	−1.74		−14.16	−1.35		−14.23	−1.49
Average	SR	0.40	0.34	0.39	0.33	0.29	0.33	0.40	0.34	0.39	0.44	0.38	0.43	0.47	0.40	0.45
	RE (%)		−14.26	−2.93		−13.39	−1.48		−14.13	−2.18		−14.70	−3.03		−14.61	−3.52

## Data Availability

The FORMOSAT-5 imagery used in this study is available from the Taiwan Space Agency (TASA) upon reasonable request and subject to institutional data access policies. RadCalNet surface reflectance data are publicly accessible at https://www.radcalnet.org/. The in situ ASD measurements collected at Railroad Valley Playa and the derived BRDF lookup table are available from the corresponding author upon reasonable request.

## Supplementary Materials

The following supporting information can be downloaded at: https://www.mdpi.com/article/10.3390/s26123719/s1, Section S1: Sensitivity of $K_{0}$ to solar and view azimuth uncertainty; Table S1: Sensitivity of *K*_0_ to solar azimuth perturbation only (Δφ*_v_* = 0); Table S2: Sensitivity of $K_{0}$ to view azimuth perturbation only (Δφ*_s_* = 0); Table S3: All non-baseline perturbation cases sorted by |ΔRAA|; Section S2: Goniometer system for multi-angle surface reflectance measurement; Figure S1: Laboratory goniometer system for multi-angle surface reflectance measurements.
