# Supplementary material for: BRDF-Corrected Vicarious Calibration of FORMOSAT-5 RSI Using RadCalNet: Quantitative Assessment and Implications for TOA Reflectance and NIRv
Source: Sensors (Basel). 2026 Jun 11;26(12):3719. doi: 10.3390/s26123719 (PMC13306664; doi:10.3390/s26123719)
Supplement: Supplementary file 1 [file sensors-26-03719-s001.zip › sensors-4333393-supplementary.pdf]

# Supplementary Material

*BRDF-Corrected Vicarious Calibration of FORMOSAT-5 RSI Using RadCalNet: Quantitative Assessment and Implications for TOA Reflectance and NIRv*

## Section S1. Sensitivity of $K_0$ to solar and view azimuth uncertainty

### Section S1.1 Motivation

Because the BRDF correction framework relies on solar and viewing geometry recorded in the image metadata, uncertainties in solar azimuth angle ($\varphi_s$) and view azimuth angle ($\varphi_v$) may propagate into the BRDF-adjusted surface reflectance, the subsequent 6S radiative transfer simulation, and ultimately the retrieved radiometric calibration coefficient $K_0$. To assess whether the calibration improvement reported in the main manuscript could be attributed to metadata uncertainty rather than BRDF correction itself, a dedicated perturbation analysis was performed.

### Section S1.2 Methodology

To quantify how robust the retrieved calibration coefficients $K_0$ are against angular uncertainty in the FS-5 metadata, we performed a 15-case perturbation study around the baseline acquisition geometry of the 12 September 2025 FS-5 overpass at Railroad Valley Playa (RVUS): $\theta_s$ = 38.04°, $\varphi_s$ = 151.93°, $\theta_v$ = 27.92°, $\varphi_v$ = 101.69°. Each case repeats the full BRDF–6S–$K_0$ pipeline. In Step 1, the BRDF correction applies perturbed azimuths as below:

$$\varphi_s^{pert} = \varphi_s + \Delta\varphi_s \qquad \text{(S1)}$$

$$\varphi_v^{pert} = \varphi_v + \Delta\varphi_v \qquad \text{(S2)}$$

Where $\Delta\varphi_v$ and $\Delta\varphi_s$ denote the applied perturbations to the solar and view azimuth angles. The perturbed azimuth angles apply to LUT to derive the spectral ratio $SR_{ratio}$ (b), which rescales the RadCalNet site reflectance. In Step 2, the same perturbed angles enter the 6S radiative transfer model for each of the five 6S band, yielding band-integrated TOA radiance$L_{TOA}$ (b). In Step 3, $K_0$(b) = DN(b) / $L_{TOA}$ (b) × F5_Gain(b) is computed on the RadCalNet Site.

$$RAA = \varphi_s - \varphi_v \qquad \text{(S3)}$$

$$\Delta RAA = \Delta\varphi_s - \Delta\varphi_v \qquad \text{(S4)}$$

where $RAA$ is the relative azimuth angle, $\varphi_s$ is the solar azimuth angle, and $\varphi_v$ is the sensor view azimuth angle. The relative azimuth angle describes the angular relationship between the illumination direction and the viewing direction and serves as a key variable controlling BRDF anisotropy. Equation (S4) shows that only the perturbation in relative azimuth angle ($\Delta RAA$) affects the BRDF-adjusted reflectance and the subsequent $K_0$ retrieval.

The perturbation set comprises: (i) baseline ($\Delta\varphi_s = \Delta\varphi_v = 0$); (ii) six $\varphi_s$-only cases with $\Delta\varphi_s \in \{\pm1°, \pm2°, \pm3°\}$ and $\Delta\varphi_v = 0$; (iii) six $\varphi_v$-only cases with $\Delta\varphi_v \in \{\pm1°, \pm2°, \pm3°\}$ and $\Delta\varphi_s = 0$; and (iv) two $RAA$ -conserved cases with $\Delta\varphi_s = \Delta\varphi_v = \pm3°$. The relative deviation is calculate as below:

$$\Delta K_0(\mathrm{b}) = \frac{K_0^{pert}(b) - K_0^{base}(b)}{K_0^{base}(b)} \times 100\% \qquad \text{(S5)}$$

which is reported per band, with baseline $K_0$ = (14.15972, 12.41233, 14.93858, 12.71746, 16.13393) for (PAN, B, G, R, NIR), respectively.

**Section S1.3 Results and Discussion**

Tables S1–S3 summarize the 15-case perturbation outcomes. Three features stand out.

First, the analysis demonstrates that $K_0$ is primarily controlled by the relative azimuth angle ($RAA$) rather than the absolute orientation of $\varphi_s$ or $\varphi_v$. Cases producing identical $\Delta RAA$ yielded identical $\Delta K_0$ values, confirming rotational invariance of both the BRDF LUT and the 6S atmospheric simulation. Second, $K_0$ exhibits an approximately linear response to $\Delta RAA$ across all five FS-5 bands, with sensitivities ranging from 0.21% to 0.24% per degree. The Blue band shows the highest sensitivity, consistent with the stronger influence of atmospheric scattering processes at shorter wavelengths. Third, even under the most extreme perturbation examined ($|\Delta RAA|$ = 3°), the corresponding $K_0$ variation remains below 0.75% for all bands.

From an error-budget perspective, this magnitude is substantially smaller than the systematic $K_0$ bias corrected by the BRDF adjustment in the main analysis, which ranges from approximately 9 to 16 % across the FS-5 bands. Thus, even the worst-case azimuth-induced uncertainty is approximately 12–21 times smaller than the BRDF-related calibration bias. This comparison indicates that metadata azimuth uncertainty is a secondary error source relative to the BRDF effect in the FS-5 $K_0$ retrieval at Railroad Valley Playa.

These results indicate that the observed improvement in $K_0$ retrieval cannot be explained by plausible metadata errors alone. Instead, the dominant source of the calibration bias originates from the mismatch between nadir-equivalent surface reflectance and the actual off-nadir observation geometry. The BRDF correction framework effectively addresses this mismatch and therefore constitutes a physically meaningful improvement to the vicarious calibration procedure. Therefore, the principal conclusions of this study are insensitive to realistic azimuth-angle uncertainties in the FS-5 metadata.

**Table S1.** Sensitivity of $K_0$ to solar azimuth perturbation only ($\Delta\varphi_v = 0$). Six cases with $\Delta\varphi_s \in \{\pm1°, \pm2°, \pm3°\}$. $\Delta RAA = \Delta\varphi_s$ by sign convention.

| $\Delta\varphi_s$ (°) | $\varphi_s^{pert}$ (°) | $\Delta RAA$ (°) | $\Delta K_0^{PAN}$(%) | $\Delta K_0^{B}$ (%) | $\Delta K_0^{G}$ (%) | $\Delta K_0^{R}$(%) | $\Delta K_0^{NIR}$(%) |
|---|---|---|---|---|---|---|---|
| −3.0 | 148.93 | −3.000 | −0.6458 | −0.7081 | −0.6743 | −0.6333 | −0.5810 |
| −2.0 | 149.93 | −2.000 | −0.4365 | −0.4765 | −0.4549 | −0.4287 | −0.3941 |
| −1.0 | 150.93 | −1.000 | −0.2215 | −0.2406 | −0.2303 | −0.2174 | −0.2008 |
| +1.0 | 152.93 | +1.000 | +0.2287 | +0.2448 | +0.2362 | +0.2250 | +0.2091 |
| +2.0 | 153.93 | +2.000 | +0.4646 | +0.4959 | +0.4795 | +0.4569 | +0.4258 |
| +3.0 | 154.93 | +3.000 | +0.7079 | +0.7532 | +0.7301 | +0.6973 | +0.6519 |

**Table S2.** Sensitivity of $K_0$ to view azimuth perturbation only ($\Delta\varphi_s = 0$). Six cases with $\Delta\varphi_v \in \{\pm1°, \pm2°, \pm3°\}$. $\Delta RAA = -\Delta\varphi_v$ because an increase in VAA reduces RAA = SAA − VAA.

| $\Delta\boldsymbol{\varphi}_v$ (°) | $\boldsymbol{\varphi}_v^{pert}$ (°) | $\Delta RAA$ (°) | $\Delta K_0^{PAN}$(%) | $\Delta K_0^{B}$ (%) | $\Delta K_0^{G}$ (%) | $\Delta K_0^{R}$(%) | $\Delta K_0^{NIR}$(%) |
|---|---|---|---|---|---|---|---|
| −3.0 | 98.69 | +3.000 | +0.7079 | +0.7532 | +0.7301 | +0.6973 | +0.6519 |
| −2.0 | 99.69 | +2.000 | +0.4646 | +0.4959 | +0.4795 | +0.4569 | +0.4258 |
| −1.0 | 100.69 | +1.000 | +0.2287 | +0.2448 | +0.2362 | +0.2250 | +0.2091 |
| +1.0 | 102.69 | −1.000 | −0.2215 | −0.2406 | −0.2303 | −0.2174 | −0.2008 |
| +2.0 | 103.69 | −2.000 | −0.4365 | −0.4765 | −0.4549 | −0.4287 | −0.3941 |
| +3.0 | 104.69 | −3.000 | −0.6458 | −0.7081 | −0.6743 | −0.6333 | −0.5810 |

**Table S3.** All 14 non-baseline perturbation cases sorted by $|\Delta RAA|$ descending. "Sens. band" denotes the band giving the largest $|\Delta K_0|$. The two $RAA$-conserved cases (both_±3) appear at the bottom with $\Delta K_0 = 0$ across all bands, confirming that absolute azimuth rotation cancels when relative azimuth is preserved.

| Case | $\Delta\boldsymbol{\varphi}_s$ (°) | $\Delta\boldsymbol{\varphi}_v$ (°) | Δ(°) | $\Delta K_0^{PAN}$(%) | $\Delta K_0^{B}$ (%) | $\Delta K_0^{G}$ (%) | $\Delta K_0^{R}$(%) | $\Delta K_0^{NIR}$(%) | max\|$\Delta K_0$\| (%) | Sens. band |
|---|---|---|---|---|---|---|---|---|---|---|
| vaa_−3 | +0.0 | −3.0 | +3.000 | +0.7079 | +0.7532 | +0.7301 | +0.6973 | +0.6519 | 0.7532 | B |
| saa_+3 | +3.0 | +0.0 | +3.000 | +0.7079 | +0.7532 | +0.7301 | +0.6973 | +0.6519 | 0.7532 | B |
| vaa_+3 | +0.0 | +3.0 | −3.000 | −0.6458 | −0.7081 | −0.6743 | −0.6333 | −0.5810 | 0.7081 | B |
| saa_−3 | −3.0 | +0.0 | −3.000 | −0.6458 | −0.7081 | −0.6743 | −0.6333 | −0.5810 | 0.7081 | B |
| vaa_−2 | +0.0 | −2.0 | +2.000 | +0.4646 | +0.4959 | +0.4795 | +0.4569 | +0.4258 | 0.4959 | B |
| saa_+2 | +2.0 | +0.0 | +2.000 | +0.4646 | +0.4959 | +0.4795 | +0.4569 | +0.4258 | 0.4959 | B |
| vaa_+2 | +0.0 | +2.0 | −2.000 | −0.4365 | −0.4765 | −0.4549 | −0.4287 | −0.3941 | 0.4765 | B |
| saa_−2 | −2.0 | +0.0 | −2.000 | −0.4365 | −0.4765 | −0.4549 | −0.4287 | −0.3941 | 0.4765 | B |
| vaa_−1 | +0.0 | −1.0 | +1.000 | +0.2287 | +0.2448 | +0.2362 | +0.2250 | +0.2091 | 0.2448 | B |
| saa_+1 | +1.0 | +0.0 | +1.000 | +0.2287 | +0.2448 | +0.2362 | +0.2250 | +0.2091 | 0.2448 | B |
| vaa_+1 | +0.0 | +1.0 | −1.000 | −0.2215 | −0.2406 | −0.2303 | −0.2174 | −0.2008 | 0.2406 | B |
| saa_−1 | −1.0 | +0.0 | −1.000 | −0.2215 | −0.2406 | −0.2303 | −0.2174 | −0.2008 | 0.2406 | B |
| both_+3 | +3.0 | +3.0 | +0.000 | +0.0000 | +0.0000 | +0.0000 | +0.0000 | +0.0000 | 0.0000 | — |
| both_−3 | −3.0 | −3.0 | +0.000 | +0.0000 | +0.0000 | +0.0000 | +0.0000 | +0.0000 | 0.0000 | — |

*Note: $\Delta K_0$ values for PAN, B, G, R, NIR are in percent (%).*

## Section S2. Goniometer system for multi-angle surface reflectance measurement

The goniometer system shown in Figure S1 was kindly provided by Dr. Si-Chee Tsay (NASA Goddard Space Flight Center) to support future multi-angle surface reflectance measurements. The system will be employed in subsequent field campaigns to construct a more complete hemispherical BRDF dataset for the Railroad Valley Playa calibration site.

The goniometer consists of a circular horizontal rail track of approximately 1.5 m diameter mounted on an adjustable tripod frame with leveling feet. A sensor arm is attached to a zenith arc that allows the ASD FieldSpec spectroradiometer to be repositioned at predefined view zenith angles. The horizontal rail enables full 360° azimuthal sampling, while the zenith arc provides coverage from nadir (VZA = 0°) to large off-nadir angles. A Spectralon reference panel is positioned at the center of the ring for simultaneous reference measurements.

To support future improvement of the BRDF lookup table (LUT) constructed in this study, the goniometer system allows the ASD FieldSpec spectroradiometer to be positioned at predefined view zenith angles (VZA) and azimuth angles across the full hemisphere. Compared to the discrete in situ multi-angle measurements conducted in the main study, goniometer-based measurements would provide systematic and continuous angular sampling, substantially increasing the node density of the BRDF LUT and reducing interpolation uncertainties at observation geometries not directly sampled in the current field campaign. The current BRDF LUT was constructed from in situ field measurements conducted during a single session at Railroad Valley Playa on 12 September 2025, which inherently limits the angular node density. Incorporating goniometer-based measurements in future work would enable the construction of a more physically rigorous BRDF model, thereby reducing uncertainties in the radiometric calibration coefficient $K_0$.

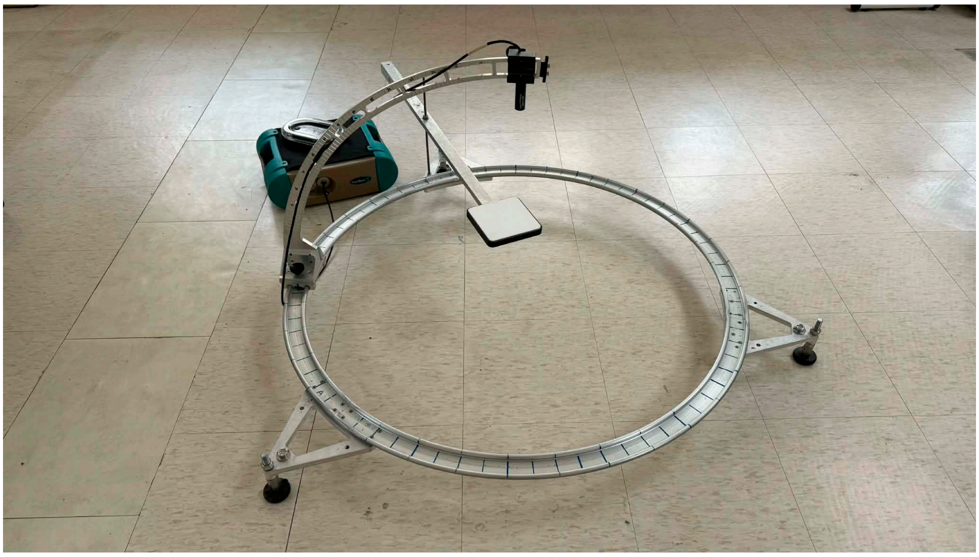

**Figure S1.** Laboratory goniometer system provided by Dr. Si-Chee Tsay (NASA Goddard Space Flight Center) for multi-angle surface reflectance measurements. The circular rail track allows the sensor to be systematically positioned at varying view zenith angles (VZA: 0°–80°) and azimuth angles (0°–360°), enabling high-density BRDF characterization of surface samples.

**Reference:**

Sandmeier, S.R. (2000). Acquisition of bidirectional reflectance factor data with field goniometers. Remote Sensing of Environment, 73(3), 257–269. https://doi.org/10.1016/S0034-4257(00)00102-2
